# Rapid Progression of Oral Leukoplakia to Oral Squamous Cell Carcinoma in an HIV-Positive Patient: A Case Report

**DOI:** 10.7759/cureus.88345

**Published:** 2025-07-20

**Authors:** Konstantinos Poulopoulos, Christina Charisi, Vasileios Zisis, Petros Papadopoulos, Evangelos Parcharidis, Eleftherios Anagnostou, Athanasios Poulopoulos

**Affiliations:** 1 Oral Medicine/Pathology, Aristotle University of Thessaloniki, Thessaloniki, GRC; 2 Pediatric Dentistry, Aristotle University of Thessaloniki, Thessaloniki, GRC; 3 Oral Medicine/Pathology, European University Cyprus, Nicosia, CYP

**Keywords:** hiv aids, oral cancers, oral leukoplakia, oral squamous cell carcinoma, potentially mailgnant disorder

## Abstract

HIV patients are at an increased risk of developing head and neck cancers, particularly due to the immunocompromised state caused by the virus. This report aims to illustrate a case of a HIV-positive patient whose oral leukoplakia rapidly progressed into oral squamous cell carcinoma. The patient was initially referred to our Department of Oral Medicine and Pathology, School of Dentistry, Aristotle University of Thessaloniki, Greece, on May 17, 2024, due to the presence of an intraoral lesion. The examination revealed a white plaque lesion on his left buccal mucosa, which was subjected to biopsy. The histopathological examination showed a moderately dysplastic leukoplakia. The patient was advised to follow bimonthly checkups, and on July 9, 2024, he was reexamined. The second examination revealed an alteration of the clinical appearance of an exophytic mass, and the patient underwent a second biopsy. This time, the lesion was proven to be an early invasive oral squamous cell carcinoma, surrounded by moderately dysplastic leukoplakia. This case signifies the necessity of regular follow-ups in patients manifesting oral potentially malignant disorders. HIV-positive individuals necessitate proactive monitoring, due to the immunosuppression, in order to detect potentially malignant disorders and malignancies at an early, treatable stage.

## Introduction

HIV status significantly impacts the natural course of oral leukoplakia, particularly in accelerating its progression to oral squamous cell carcinoma (OSCC) through immunosuppression and chronic inflammation. HIV immunodeficiency predisposes individuals to the development of oral lesions such as leukoplakia but also compromises the oral mucosa’s ability to repair and defend against malignant transformation [1,2]. Dysplastic leukoplakia refers to a potentially malignant disorder of the oral mucosa where the epithelial cells exhibit changes in their appearance and dysplasia under the microscope. These changes suggest an increased risk of developing into oral cancer, which depends on the degree of dysplasia (severe> moderate> mild). This is exacerbated by chronic inflammation, which promotes cellular proliferation and genetic instability within the oral epithelium [3]. Moreover, the presence of additional risk factors-such as tobacco use, alcohol consumption, and human papillomavirus (HPV) infection-may synergistically increase the likelihood that oral leukoplakia will progress to OSCC in HIV-positive individuals [4,5]. HIV infection is in itself an independent risk factor for oral squamous cell carcinoma, suggesting a direct link between HIV-driven immunosuppression and malignant transformation in oral potentially malignant disorders [6]. The risk of malignant transformation in oral potentially malignant lesions among HIV-positive individuals necessitates regular monitoring, and follow-ups are essential to ensure early detection. The regular oral screening is biannual [7]. Moreover, if a patient develops oral cancer, the follow-up period should be extended to account for the heightened risk of recurrence [7]. This report aims to illustrate a case of HIV HIV-positive patient whose oral leukoplakia rapidly progressed into oral squamous cell carcinoma.

## Case presentation

**Figure 1:**
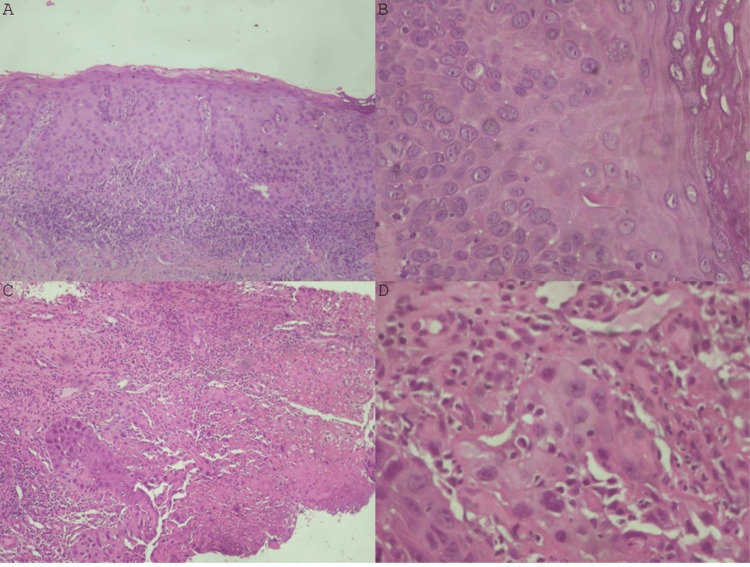
Histopathological examinations First histological examination of the oral leukoplakia: (A) Moderate dysplasia of squamous cell epithelium, with loss of polarity, moderate nuclear atypia, and mitoses in the lower two-thirds of the epithelium (hematoxylin-eosin stain X100); (B) Moderate dysplasia of squamous cell epithelium, without invasion of lamina propria (hematoxylin-eosin stain X400). Second histological examination of the oral squamous cell carcinoma: (C) Severe dysplasia and focal invasive squamous cell carcinoma (hematoxylin-eosin stain X100). (D) Severe dysplasia and focal invasive squamous cell carcinoma, with invasive growth of anomalous solid nests of neoplastic cells (hematoxylin-eosin stain X400).

**Figure 2:**
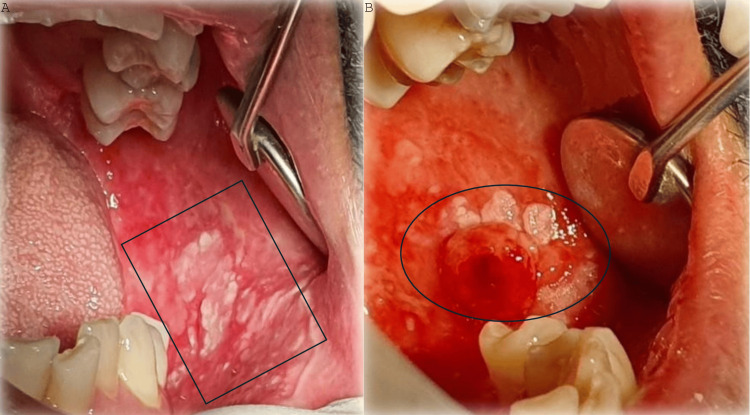
Sequential clinical images (A) Initial clinical appearance of the moderately dysplastic leukoplakia (squared area). (B) Clinical appearance of the oral squamous cell carcinoma approximately eight weeks after the initial appearance (circled area).

A 57-year-old male patient was initially referred to the Department of Oral Medicine and Pathology, School of Dentistry, Aristotle University of Thessaloniki, Greece, on May 16, 2024, due to the presence of an intraoral lesion. The medical history included an HIV-positive status since 1991, which the patient had under control. In particular, the past blood examinations showed that the virus load was undetectable, whereas the CD4 count was above 450 cells/mm³. The therapeutic regimen for the time being included the administration of BIKTARVY (Bictegravir/tenofovir alafenamide/emtricitabine (BIC/TAF/FTC)), one tab per day. The examination revealed a white plaque lesion on his left buccal mucosa, which was subjected to biopsy. The patient did not report any symptoms and could not remember when the lesion commenced. Furthermore, the patient did not mention any relevant habits or environmental habits, such as tobacco or alcohol consumption. The histopathological examination showed a moderately dysplastic leukoplakia (Figure 1A-1B) (Figure 2A).

The patient was advised to follow bimonthly checkups, and on July 9, 2024, he was reexamined. The second examination revealed an alteration of the clinical appearance of an exophytic mass, and the patient underwent a second biopsy. This time, the lesion was proven to be an early invasive oral squamous cell carcinoma, surrounded by the moderately dysplastic leukoplakia (Figure 1C-1D) (Figure 2B).

## Discussion

**Figure 3:**
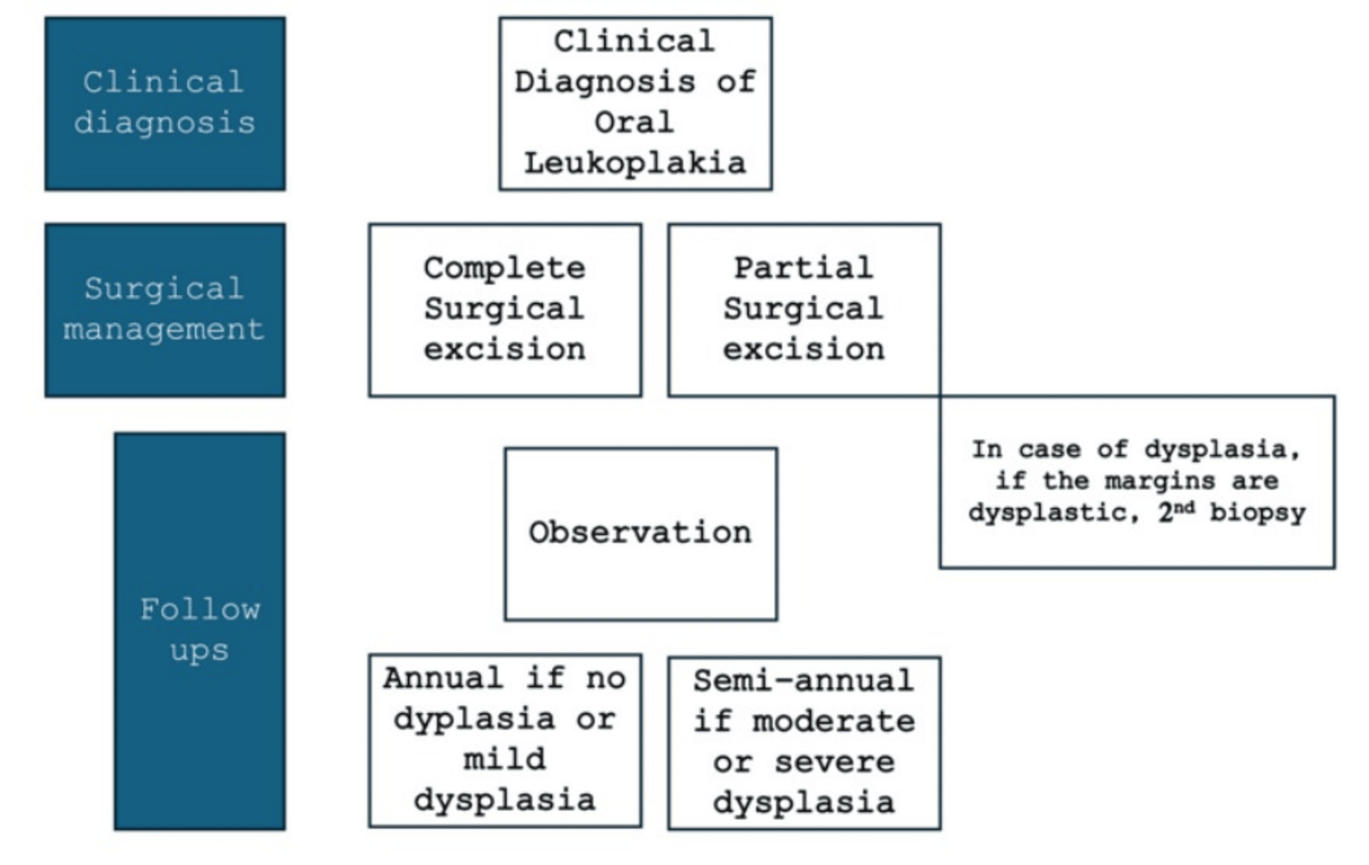
Flowchart of management of oral leukoplakia

OSCC in HIV-positive patients often presents with distinctive clinical features reflecting the underlying immunosuppression. Figure 3 depicts the general flowchart of oral leukoplakia management.

The most common symptoms include persistent ulceration, pain, swelling, and, less often, bleeding or induration [8,9]. These symptoms are not only indicative of OSCC but may also be confounded by other HIV-associated oral lesions, such as Kaposi's sarcoma, non-Hodgkin lymphoma, fungal or viral infections [10,11]. Furthermore, HIV-positive individuals are predisposed to more aggressive disease progression, with a higher likelihood of presenting at advanced stages and with lesions located at high-risk sites such as the tongue and floor of the mouth [12,13]. A recent systematic review and meta-analysis deduced that the OL tended to develop into OSCC at 7.2% [14]. In people living with HIV, a threefold increased incidence of head and neck squamous cell carcinoma is to be expected, and at a significantly younger age, as well [15]. Our case constitutes such an example of aggressive progression since the potentially malignant lesion developed into full-fledged OSCC in less than two months. The clinical progression may be further enhanced by co-infections with oncogenic viruses like Epstein-Barr virus (EBV), which is detected in a significant percentage of oral tumors among HIV-positive patients [16]. Healthcare access and regular screening may influence the timing of OSCC manifestation, mitigating the deviation between HIV-positive and HIV-negative individuals [17]. The efficient primary care contributes to early detection and intervention, offsetting the biological disadvantages imposed by HIV status [17]. The histopathology of HIV-associated OSCC affects tumor morphology and progression [4]. Moderate epithelial dysplasia and hyperkeratosis are commonly observed in biopsy specimens from affected sites such as the soft palate, which aligns with the increased prevalence of exophytic, elevated lesions exhibiting a papillary architecture in these patients [13,18]. Furthermore, molecular alterations in key regulatory proteins and genes related to cell proliferation and apoptosis are observed in HIV-associated OSCC [19]. The impact of tobacco and alcohol remains significant because they may exert a synergistic detrimental effect combined with immunosuppression, accelerating the onset and progression of OSCC [20]. Additionally, the human papillomavirus (HPV) status is more commonly positive in HIV-positive patients, and these HPV-related oncogenic strains contribute substantially to the risk of malignant transformation [20]. Early initiation of antiretroviral therapy (ART) is the optimal approach for achieving comprehensive immune restoration, preventing AIDS-related complications [21]. ART functions by suppressing HIV replication, leading to increased CD4 cell counts and a reduction in systemic inflammation [22,23]. However, immune restoration after ART can be unpredictable; some individuals experience incomplete normalization of immune function, leaving them susceptible to ongoing inflammation and the associated morbidity, including oral malignancies and other co-morbidities [21,23]. The five-year survival rates remain poor due to the aggressive nature of the disease and the limited long-term effectiveness of both surgical and non-surgical interventions [24]. Chemoradiotherapy as a primary modality has demonstrated improved progression-free and overall survival when compared with radiotherapy by itself [24]. The results of the combined approach with surgery remain mixed and without definitive conclusions. Two-year survival rates for patients treated with chemoradiotherapy can be relatively favorable, ranging from 72.9% to 82%, yet these percentages plummet over time [24]. Recurrence remains a major concern, with re-recurrence rates after locoregional treatment ranging from 35% to 72% [24]. The management of locoregional recurrence influences the post-recurrence survival: patients receiving active treatment for recurrence demonstrate better survival rates-median post-recurrence survival of 19.9 months compared to just 4.0 months for those receiving only supportive care [24].

Regarding the need for rigorous follow-ups, there is no international consensus in effect [25]. The point of post-therapeutic surveillance is undisputed; the exact schedule is still under discussion. The intervals tend to be shorter in the first year, with relative expansion in the second year, and fewer visits in the third year [25]. The majority of references agree that follow-up beyond the third year may be deemed an unnecessary waste of medical resources and time. Finally, there is no consensus on the intervals of imaging examinations.

## Conclusions

This case signifies the necessity of regular follow-ups in patients manifesting oral potentially malignant disorders. HIV-positive individuals necessitate proactive monitoring, due to the immunosuppression, in order to detect potentially malignant disorders and malignancies at an early, treatable stage. There is a critical need for more robust, long-term strategies to minimize recurrence and improve survival, as well as further research into optimizing treatment selection in this vulnerable population. Possibly, a stricter follow-up protocol should be implemented in such cases, three or even four times per annum.
